# Loss of p16 does not protect against premature ovarian insufficiency caused by alkylating agents

**DOI:** 10.1186/s12884-023-05476-x

**Published:** 2023-03-08

**Authors:** Fei Liu, Qin Wan, Pengfei Liu, Dengshun Miao, Xiuliang Dai, Li Chen

**Affiliations:** 1 The Center for Reproductive Medicine, Changzhou Maternal and Child Health Care Hospital, Changzhou Medical Center, Nanjing Medical University, Changzhou, Jiangsu China; 2 Kebiao Medical Testing Center, Changzhou, Jiangsu China; 3 The Research Center for Aging, Affiliated Friendship Plastic Surgery Hospital of Nanjing Medical University, Nanjing Medical University, Nanjing, China

**Keywords:** Premature ovarian insufficiency, Alkylating agents, p16 deficiency, Folliculogenesis

## Abstract

**Background:**

Chemical agents such as alkylating agents (AAs) that are commonly used for the treatment of cancer cause great damage to the ovaries, thereby significantly increasing the risk of premature ovarian insufficiency (POI). However, the exact molecules underlying AA-induced POI remain largely obscure. Upregulation of the p16 gene may contribute to the progression of POI. As yet, no in vivo data from p16-deficient (KO) mice are available to demonstrate a critical role of p16 in POI. In the present study, we employed p16 KO mice to investigate whether loss of p16 could protect against POI caused by AAs.

**Methods:**

WT mice and their p16 KO littermates received a single dose of BUL + CTX to establish an AA-induced POI mouse model. One month later, oestrous cycles were monitored. Three months later, some of the mice were sacrificed to collect sera for measurements of hormone levels and ovaries for measurements of follicle counts, the proliferation and apoptosis of granulosa cells, ovarian stromal fibrosis and vessels. The remaining mice were mated with fertile males for the fertility test.

**Results:**

Our results showed that treatment with BUL + CTX significantly disrupted the oestrous cycles, increased the levels of FSH and LH while decreasing the levels of E2 and AMH, decreased the counts of primordial follicles and growing follicles while increasing the counts of atretic follicles, reduced the vascularized area in the ovarian stroma, and decreased fertility. All of these results were comparable between WT and p16 KO mice treated with BUL + CTX. In addition, ovarian fibrosis was not increased significantly in WT and p16 KO mice treated with BUL + CTX. Growing follicles with normal appearance had normally proliferating granulosa cells (without apparent apoptosis).

**Conclusion:**

We concluded that genetic ablation of the p16 gene did not attenuate ovarian damage or help preserve the fertility of mice challenged by AAs. This study demonstrated for the first time that p16 is dispensable for AA-induced POI. Our preliminary findings suggest that targeting p16 alone may not preserve the ovarian reserve and fertility of females treated with AAs.

**Supplementary Information:**

The online version contains supplementary material available at 10.1186/s12884-023-05476-x.

## Introduction

Premature ovarian insufficiency (POI), diagnosed mainly by elevated FSH (> 40 IU/L) and amenorrhea before 40 years old, affects 1 in 100 females[1]. Due to very poor ovarian reserve, it is difficult for females with POI to become pregnant naturally or with the aid of assisted reproductive technology. In addition to infertility, POI also increases other health risks, including osteoporosis, cardiovascular disease, and earlier mortality [2–4]. Several causes of POI have been reported, including genetic alterations, ovarian surgery, radio- or chemotherapy, environmental factors, viral infections, and metabolic and autoimmune diseases [5]. Of them, chemical agents such as alkylating agents (AAs) that are commonly used for the treatment of cancer cause great damage to the ovaries, thereby significantly increasing the risk of POI [6]. However, the exact molecules underlying AA-induced POI remain largely obscure.

p16, a well-known cell cycle inhibitor encoded by the CDKN2A gene locus (which also encodes p19), plays an important role in arresting the cell cycle and maintaining the state of cell senescence [7, 8]. p16 has been widely used as a biomarker reflecting cell senescence [9]. Targeting p16 has been demonstrated to be an effective way to lengthen the healthy lifespan and prevent multiple diseases in mice, including emphysema, tubulointerstitial injury and osteoporosis [10–13]. Recent studies have indicated a role for p16 in promoting the development of POI. It has been observed that p16 is significantly upregulated in the ovaries of several mouse models of POI, including a natural ovarian ageing mouse model and cyclophosphamide-, d-galactose- and consecutive superovulation-induced POI mouse models [14–18]. The agents that were reported to exert a protective effect against ovarian insufficiency, including metformin, moxibustion and curcumin, could significantly downregulate the expression of p16 in the ovaries [14, 15, 17]. In addition, a study conducted by Xiong et al. indicated that p16 was the ultimate effector that mediated cyclophosphamide-induced ovarian failure in mice [16]. These studies suggest that the upregulation of p16 may be a universal mechanism involved in promoting POI, including AA-induced POI.

However, the abovementioned studies indicated rather than demonstrated a role of p16 in promoting POI. As yet, no in vivo data from p16-deficient (KO) mice are available to demonstrate a critical role of p16 in POI. Without this evidence from KO mice, it remains unknown whether p16 could be a target for preserving ovarian function and fertility in females treated with chemical agents.

To address this issue, we employed p16-deficient female mice in the present study. p16 KO and wild-type (WT) female mice were treated with busulfan (BUL) in combination with cyclophosphamide (CTX) to establish a POI mouse model as previously described [19]. The reproductive parameters, including oestrous cycles, hormone levels, fertility, and follicle counts, were measured. In addition, cell proliferation and apoptosis and ovarian stromal vessels and fibrosis were compared among WT and KO mice treated with/without BUL + CTX.

## Materials and Methods

### Animals and treatment

All mice were housed in an SPF laboratory characterized by a 12 h light/dark cycle, 23 ± 1 °C and 40–60% humidity, with free access to food and water. p16 heterozygous (p16^+/−^) mice on FVB N2 background were provided by Dr. Dengshun Miao from Nanjing Medical University. Male and female p16^+/−^ were mated to generate WT and p16 KO mice for further experiments. Eight-week-old WT and p16 KO mice received a single dose of BUL (Sigma, China, 20 mg/kg) and CTX (Selleck, China, 120 mg/kg) by intraperitoneal injection as previously described [19]. One month after administering AAs, the oestrous cycle for each mouse was monitored for 34 consecutive days. Three months later, except for 6 mice in each group for the fertility test, the remaining mice were sacrificed by cervical dislocation. Serum and ovaries were collected for further analysis. Twenty-four hours before sacrifice, 3 mice in each group were injected with BrdU solution (Sigma, China) at a dose of 50 mg/kg. All animal procedures and experiments were approved by the ethics committee of Changzhou Maternal and Healthy Care Hospital.

### Fertility test

One female mouse and one WT fertile male mouse was mated in one cage for one month. Fertile male mice were confirmed by mating experiments. After one month or female pregnancy, the male mouse was removed from the cage. Female mice without pregnancy were observed for another month. The pregnant mice and litter size were recorded.

### Oestrous cycle detection

A vaginal smear was performed at approximately 9:00 am. Briefly, 10 µL of saline was pushed into the vagina and drawn with a micropipette 3 times. Then, saline containing exfoliated vaginal cells was smeared on a slide. After air drying, the smear was fixed with 75% ethanol. HE staining was performed to evaluate the cell types. The oestrous cycle was read and recorded for each mouse.

### Hormone level assay

Serum levels of AMH, FSH, LH and E2 were determined by ELISA kits. ELISA kits including anti-FSH (RJ-17,024), anti-E2 (RJ-17,014) and anti-LH (RJ17209) were used. All ELISA kits were purchased from Shanghai Renjie Biological Company. All procedures were performed strictly according to the instructions provided by the manufacturer.

### Follicle counts

Paraffin-embedded ovaries were serially sectioned. The first section containing ovarian tissue was collected. Every six sections, another section was collected until 20 sections were collected. HE staining was performed to evaluate the morphology of follicles. The criteria for the classification of follicles are as follows: primordial follicle, the oocyte was enclosed by a layer of squamous granulosa cells; primary follicle, the oocyte was enclosed by cuboidal granulosa cells; secondary follicle, the oocyte was enclosed by 2 or more layers of granulosa cells; and antral follicle, an antrum cavity was present. We avoided recording the same follicle more than once.

### Immunohistochemistry

After dewaxing and hydration, ovarian sections were immersed in citrate solution for antigen retrieval by a high-pressure method. After naturally cooling down the citrate solution, the slides were removed, and ovarian sections were incubated in 3% H_2_O_2_ for half an hour at room temperature (RT). Then, ovarian sections were incubated in 10% donkey serum for 1 h at RT. After this, ovarian sections were incubated with primary antibodies, including anti-p16 (YM0494, Immunoway, China), anti-cleaved caspase 3 (GB11532, Servicebio, China), anti-αSMA (67735-1, Proteintech, China), anti-CD31 (#77699, Cell Signaling Technology, China) and anti-BrdU antibodies (B2531, Millipore, China), at 4 °C overnight. Then, ovarian sections were incubated with HRP-labelled goat anti-mouse and anti-rabbit antibodies (A0208 and A0216, Beyotime Biotechnology, China) for 1 h at RT. Positive staining was visualized by DAB staining. For BrdU staining, additional steps were needed. After dewaxing and hydration, the sections were incubated with diluted hydrochloric acid (1:5; v:v) for 30 min at RT, followed by direct incubation with a boric acid solution for 20 min at RT. The remaining steps were the same as those of conventional immunohistochemistry.

### Picrosirius red staining (PRS)

After dewaxing and hydration, ovarian sections were stained with picrosirius red solution (R21890, Saint-bio, China) at RT for 1 h. Then, ovarian sections were dehydrated using graded ethanol and became transparent by xylene. The slides were mounted, and photos were taken.

### Statistical analysis

All values are presented as the mean ± SD. One-way ANOVA followed by post hoc Tukey’s honestly significant difference test was used to compare data among groups. SPSS software (ver. 18.0; SPSS, Inc., Chicago, IL, USA) was used for the statistical analyses.

## Results

### Effect of p16 deletion on the oestrous cycles of mice treated with BUL + CTX

**Fig. 1:**
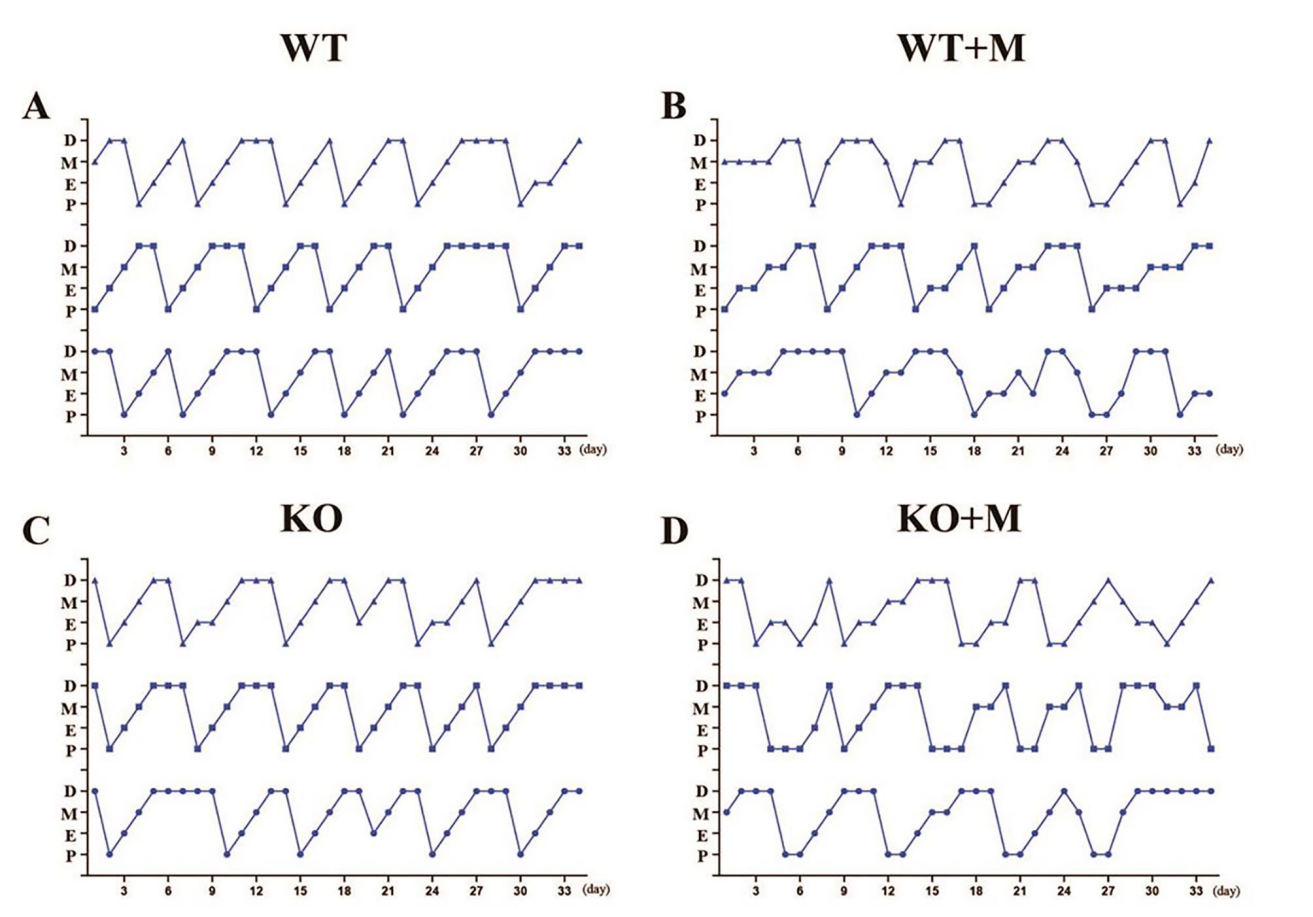
Effect of p16 deletion on the oestrous cycles of mice treated with BUL + CTX. One month after administering AAs, the oestrous cycle was monitored every day for 34 consecutive days for (**A**) untreated WT mice, (**B**) WT mice treated with BUL + CTX (WT + M), (**C**) untreated p16 KO mice and (**D**) p16 KO mice treated with BUL + CTX (KO + M). *n* = 6 mice for each group

Immunostaining for p16 showed that p16 was significantly upregulated in the ovaries of mice following treatment with BUL + CTX (Sup. Figure 1). To investigate whether p16 serves as a critical molecule that mediates the development of POI caused by BUL + CTX, we used WT and p16 KO female mice in the present study. Female mice treated with BUL + CTX showed a disrupted distribution of oestrous cycles. Therefore, we first examined the impact of p16 deletion on oestrous cycles in mice treated with BUL + CTX. Female WT mice and their p16 KO littermates received a single injection of BUL + CTX. One month later, the oestrous periods were monitored every day for 34 consecutive days. Our results showed that both WT mice and their p16 KO littermates treated with BUL + CTX showed irregular oestrous cycles, whereas untreated WT and p16 KO mice showed regular oestrous cycles (Fig. 1A-D). In addition, no apparent difference in oestrous cycles was observed between WT mice and their p16 KO littermates treated with BUL + CTX (Fig. 1A-D). These results indicated that p16 deficiency had no effect on the oestrous cycles of mice treated with BUL + CTX.

### Effect of p16 deletion on the hormone levels of mice treated with BUL + CTX

**Fig. 2:**
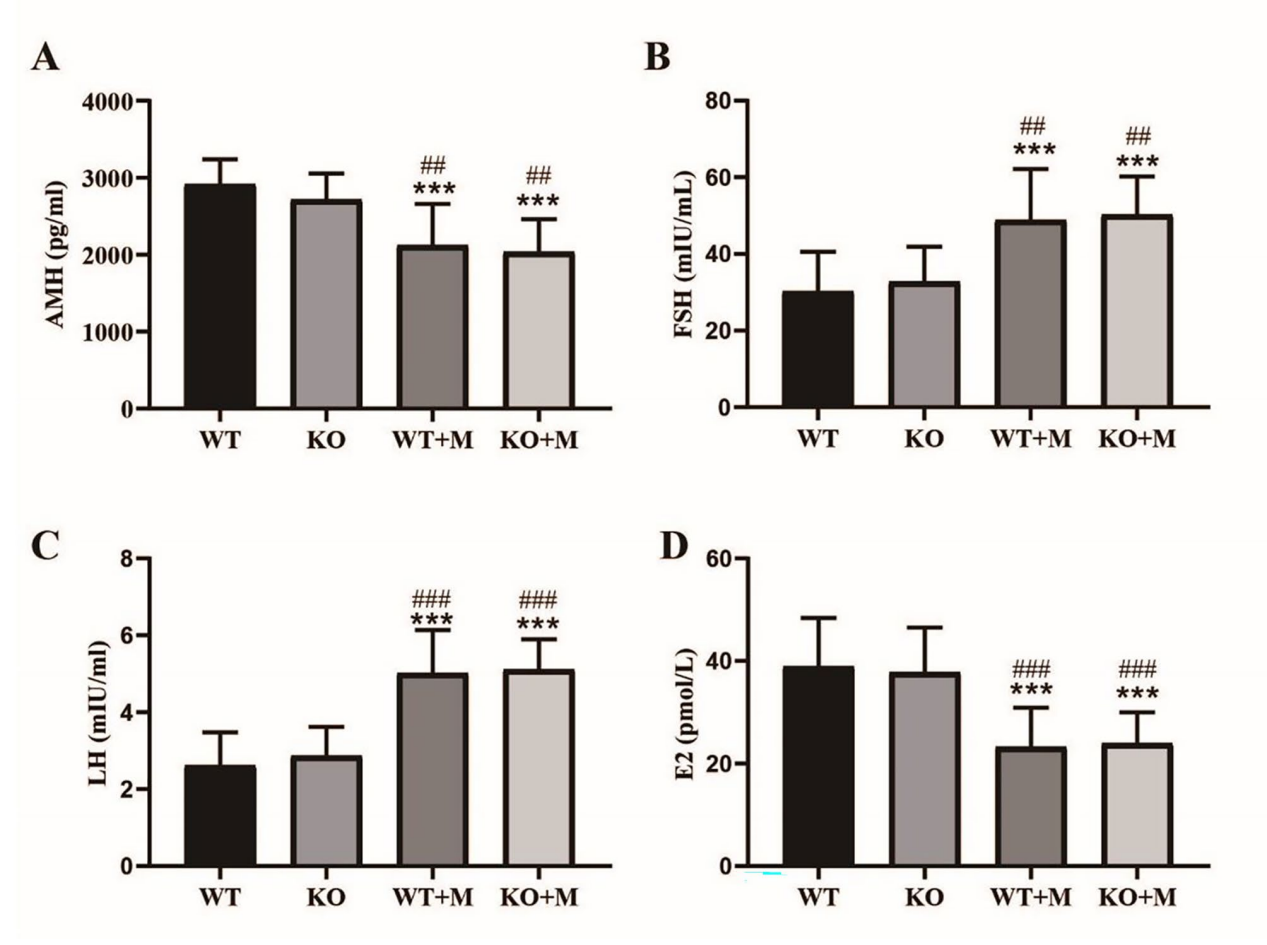
Effect of p16 deletion on the hormone levels of mice treated with BUL + CTX. Three months after administering AAs, the sera of mice were collected to detect the levels of (**A**) AMH, (**B**) FSH, (**C**) LH and (**D**) E2. Compared with WT mice: ^***^P < 0.001. Compared with p16 KO mice: ^##^*P* < 0.01; ^###^*P* < 0.001. *n* = 12 mice for each group. WT: WT mice; WT + M: WT mice treated with BUL + CTX; KO mice: untreated p16 KO mice; KO + M: p16 KO mice treated with BUL + CTX.

AMH is a good serum marker reflecting ovarian reserve. Elevated serum FSH and LH levels and decreased serum E2 levels also indicate a decline in ovarian reserve. The ovarian follicle-depleting effect of AAs has been demonstrated in rodents and humans [20]. Therefore, we measured the serum hormone levels in mice to investigate whether p16 KO could improve the hormone levels in mice treated with BUL + CTX. Three months after administering drugs, serum samples were collected separately for measurement of hormone levels. The results showed that untreated WT mice and their p16 KO littermates showed comparable hormone levels, including AMH, FSH, E2 and LH (Fig. 2A-D). As expected, treatment with BUL + CTX significantly reduced the levels of AMH and E2 and elevated the levels of FSH and LH (Fig. 2A-D). In addition, p16 KO mice treated with BUL + CTX showed hormone levels comparable to those of their WT counterparts (Fig. 2A-D). These results indicated that p16 deficiency had no effect on the hormone levels of mice treated with BUL + CTX.

### Effect of p16 deletion on the follicle number at each stage of mice treated with BUL + CTX

**Fig. 3:**
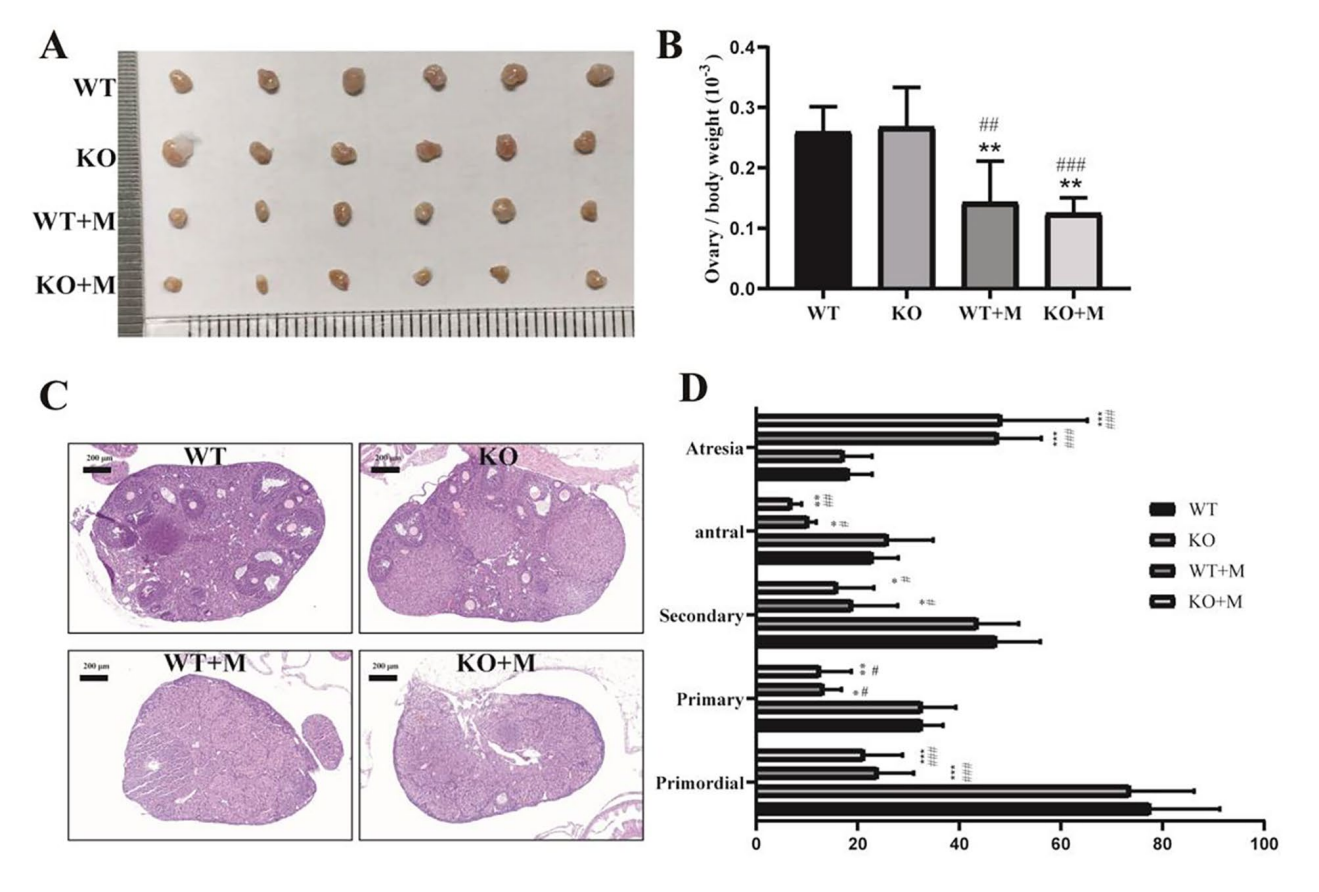
Effect of p16 deletion on the follicle number at each stage of mice treated with BUL + CTX. Three months after administering AAs, the ovaries were removed. (**A**) Size of ovaries. n = 6 for each group. (**B**) Ratio of ovarian weight to body weight. *n* = 6 for each group. (**C**) Representative photos of ovarian sections stained with HE, 6 X, n = 3 for each group. (**D**) Statistical graph of primordial and growing follicle counts. Compared with WT mice: ^*^P < 0.05; ^**^P < 0.01; ^***^P < 0.001. Compared with p16 KO mice: ^#^*P* < 0.05; ^##^*P* < 0.01; ^###^*P* < 0.001. WT: WT mice; WT + M: WT mice treated with BUL + CTX; KO mice: untreated p16 KO mice; KO + M: p16 KO mice treated with BUL + CTX.

The similar hormone levels in WT mice and their p16 KO littermates treated with BUL + CTX indicated that p16 deficiency had no impact on the decreased ovarian reserve of mice treated with BUL + CTX. To directly investigate this issue, the ovaries were removed from mice three months after administering AAs, and ovarian size and weight were recorded. Then, H&E staining was performed on consecutive ovarian sections for the purpose of follicle counts. Our results showed that untreated WT mice and their p16 KO littermates showed comparable ovarian size and ratio of ovarian weight to body weight (Fig. 3A-B). Treatment with BUL + CTX significantly reduced the ovarian size and the ratio of ovarian weight to body weight of both WT and p16 KO mice (Fig. 3A-B). However, p16 KO mice treated with BUL + CTX showed a comparable ovarian size and ratio of ovarian weight to body weight to their WT counterparts (Fig. 3A-B), indicating inactive folliculogenesis in both WT and p16 KO ovaries treated with BUL + CTX. The follicle counts showed that untreated WT mice and their p16 KO littermates showed a comparable number of follicles, including primordial follicles, primary follicles, secondary follicles, antral follicles and atretic follicles (Fig. 3C-D). Treatment with BUL + CTX significantly reduced the counts of primordial follicles and growing follicles while increasing the counts of atretic follicles in both WT and p16 KO mice (Fig. 3C-D). However, p16 KO mice treated with BUL + CTX showed comparable follicle counts in each stage, including atretic follicles, with their WT counterparts (Fig. 3C-D). In addition, we found that many primary follicles (and some secondary follicles) without oocytes (“empty follicles”) were present in the ovarian cortex of mice treated with BUL + CTX (Sup. Figure 2). No significant difference in the number of “empty follicles” was observed between WT and p16 KO mice treated with BUL + CTX (Sup. Figure 2). In contrast, untreated WT and p16 KO mice showed few “empty follicles” (Sup. Figure 2). These results may imply overactivation of primordial follicles and subsequent loss of eggs, leading to accelerated exhaustion of the pool of primordial follicles. Collectively, these results indicated that p16 deficiency had no effect on follicle counts in each stage of mice treated with BUL + CTX.

### Effect of p16 deletion on ovarian cell proliferation and apoptosis in mice treated with BUL + CTX

**Fig. 4:**
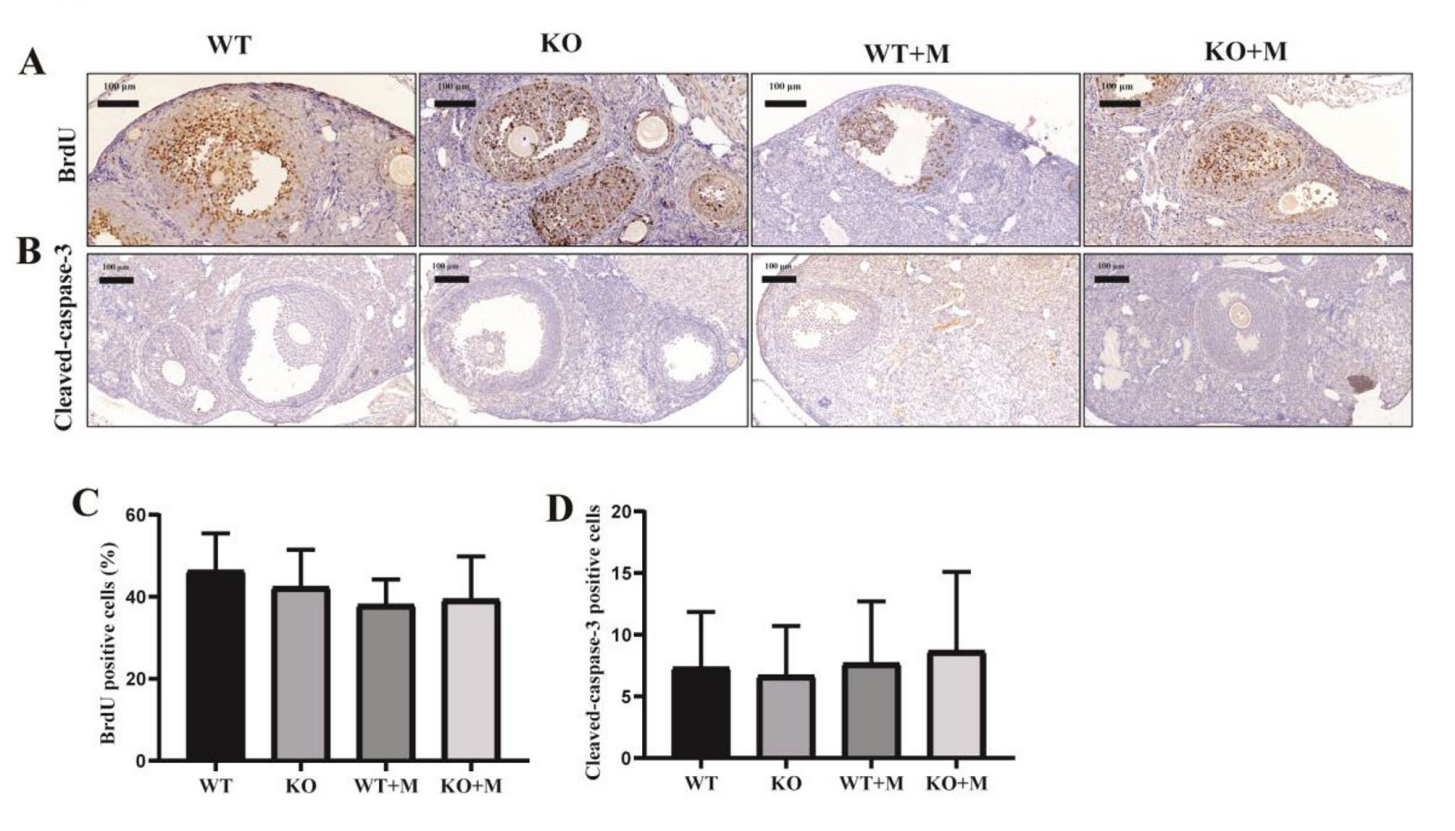
Effect of p16 deletion on the proliferation and apoptosis of ovarian granulosa cells. Three months after administering AAs, the ovaries were removed and sectioned. Representative photos of ovarian sections stained with (**A**) anti-BrdU antibody, 240X, and (**B**) anti-cleaved caspase-3 antibody, 200X, *n* = 3 mice in each group. (**C**) The percentage of BrdU positive cells, *n* = 3 mice in each group. (**D**) The number of cleaved-caspase-3 positive cells. WT: WT mice; WT + M: WT mice treated with BUL + CTX; KO mice: untreated p16 KO mice; KO + M: p16 KO mice treated with BUL + CTX.

p16 acts as a well-known cell cycle inhibitor and a widely accepted marker of cell senescence [8]; therefore, we investigated the effects of p16 deficiency on cell proliferation. BrdU incorporation assays showed that the number of proliferating granulosa cells within follicles was comparable among groups (Fig. 4A, C). Immunostaining for cleaved caspase-3 in ovarian sections showed that no apparent caspase-3-positive cells within follicles were observed among the groups (Fig. 4B, D). These results indicated a certain number of relatively healthy follicles in WT and p16 KO mice treated with BUL + CTX.

### Effect of p16 deletion on ovarian stromal abnormalities in mice treated with BUL + CTX

**Fig. 5:**
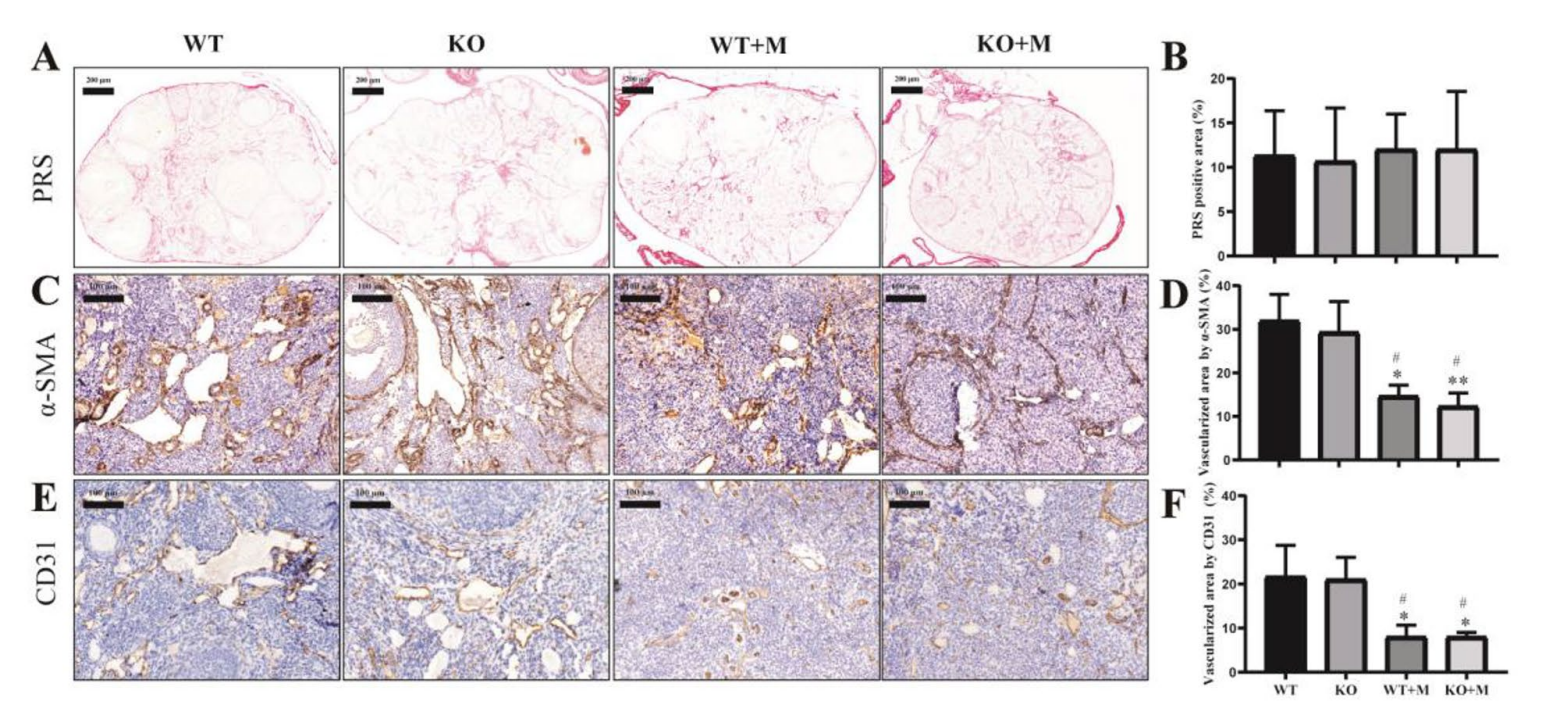
Effect of p16 deletion on ovarian stromal abnormalities in mice treated with BUL + CTX. Three months after administering AAs, the ovaries were removed and sectioned. Representative photos of ovarian sections stained with (**A**) picrosirius red, 6X, and (**B**) The percentage of PRS positive area. *n* = 3 mice in each group. Representative photos of ovarian sections stained with (**C**) α-SMA antibody, 200X, and (**D**) the percentage of vascularized area visualized by α-SMA, *n* = 3 mice in each group. Representative photos of ovarian sections stained with (**E**) CD31 antibody, 200X, and (**F**) the percentage of vascularized area visualized by CD31, *n* = 3 mice in each group. Compared with WT mice: ^*^P < 0.05; ^**^P < 0.01. Compared with p16 KO mice: ^#^*P* < 0.05.WT: WT mice; WT + M: WT mice treated with BUL + CTX; KO mice: untreated p16 KO mice; KO + M: p16 KO mice treated with BUL + CTX.

Ovarian stromal abnormalities, including ovarian fibrosis and decreased density of ovarian vessels, have been reported in females treated with chemicals [21]. In the present study, we checked the status of ovarian fibrosis and the morphology of blood vessels to determine whether p16 deficiency may have an effect on possible ovarian stromal alterations caused by BUL + CTX. It has been demonstrated that PRS staining can specifically label fibrosis in the ovarian stroma in mice of reproductive age [22]. Our results showed no significant difference in PRS-positive staining among the groups (Fig. 5A, B). α-SMA, which is a marker of peri-endothelial cells, such as pericytes and vascular smooth-muscle cells, can be used to visualize the angioarchitecture in tissues [23]. Our results showed that the vascularized area in the ovaries was significantly reduced in both WT and p16 KO mice treated with BUL + CTX compared to untreated mice (Fig. 5C, D). In addition, there was no significant difference in the ovarian vascularized area between WT and KO mice treated with BUL + CTX (Fig. 5C, D). Consistently, CD31 (a marker of vascular endothelial cells) immunostaining also showed a significant reduction in the vascularized area in the ovarian stroma of mice treated with BUL + CTX compared to untreated mice (Fig. 5E, F). Similarly, there was no significant difference in the ovarian vascularized area visualized by CD31 between WT and KO mice treated with BUL + CTX (Fig. 5E, F). In addition, dot distributions of CD31-positive single or several cells in the ovarian stroma were observed in WT and p16 KO mice treated with BUL + CTX, indicating activated neovascularization (Fig. 5E, F). These results indicated that p16 deficiency had no effect on ovarian stromal abnormalities in mice caused by BUL + CTX.

### Effect of p16 deletion on the fertility of mice treated with BUL + CTX

**Fig. 6:**
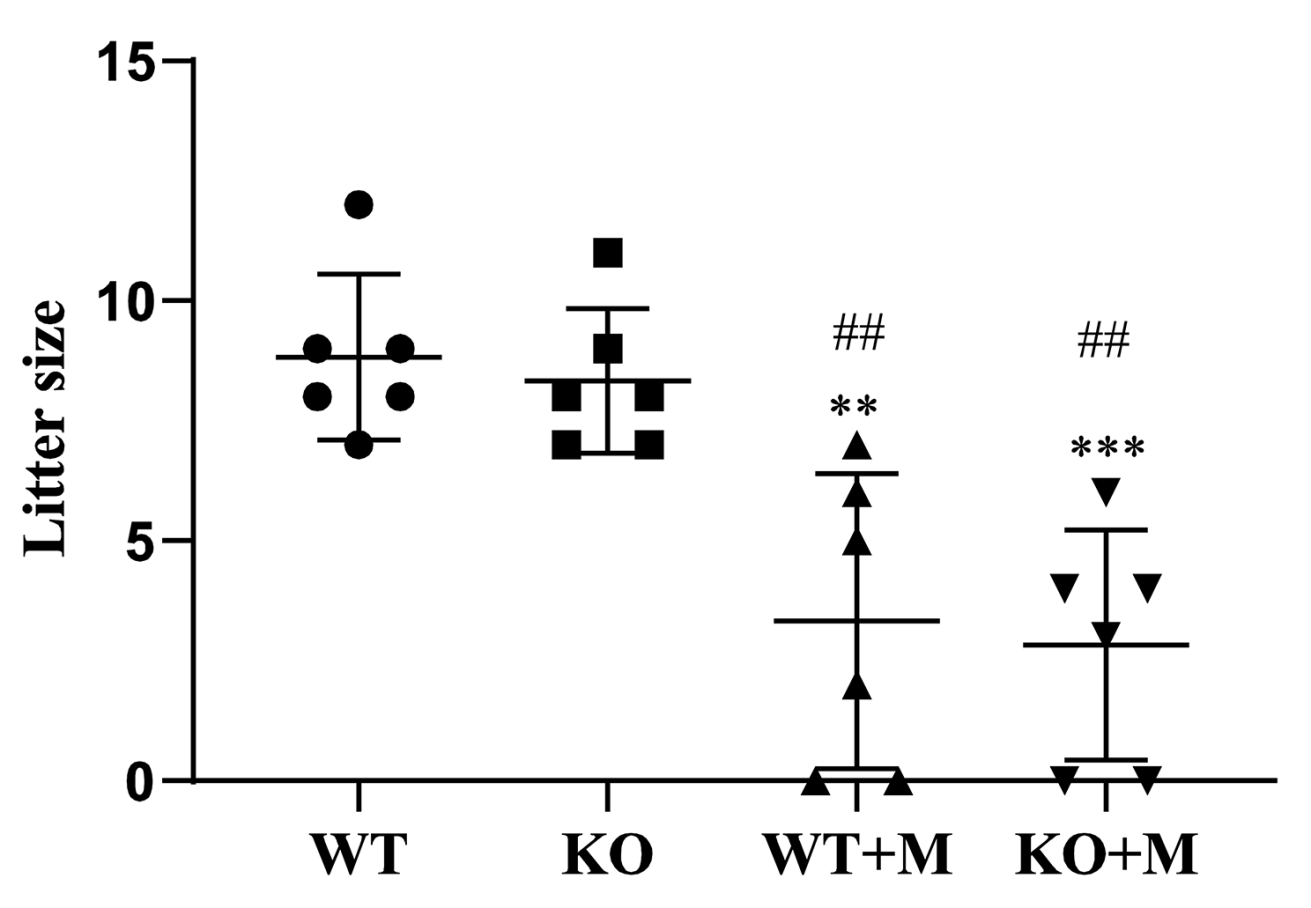
Effect of p16 deletion on the fertility of mice treated with BUL + CTX. Three months after administering AAs, the females were mated with fertile males. The fertility and litter size in each group. Compared with WT mice: ^**^P < 0.01; ^***^P < 0.001. Compared with p16 KO mice: ^##^*P* < 0.01. WT: WT mice; WT + M: WT mice treated with BUL + CTX; KO mice: untreated p16 KO mice; KO + M: p16 KO mice treated with BUL + CTX.

Finally, we tested whether p16 deficiency may affect fertility in mice treated with BUL + CTX. Three months after administering BUL + CTX, untreated and treated WT and p16 KO female mice were mated with fertile male mice for one month. We found that all untreated WT and KO mice became pregnant and gave birth to offspring (Fig. 6). The average litter size for untreated WT mice was 9, while the average litter size for untreated KO mice was 8 (Fig. 6). There was no significant difference in litter size between untreated WT and p16 KO mice (Fig. 6). In contrast, 2 out of 6 WT and p16 KO female mice treated with BUL + CTX were infertile (Fig. 6), while 4 out of 6 WT and p16 KO female mice treated with BUL + CTX became pregnant and gave birth to offspring (Fig. 6). The average litter size for fertile WT mice treated with BUL + CTX was 5, while the average litter size for fertile p16 KO mice treated with BUL + CTX was 4 (Fig. 6). There was no significant difference in litter size between WT and p16 KO mice treated with BUL + CTX (Fig. 6). These results indicated that p16 deficiency had no effect on preserving the fertility of mice treated with BUL + CTX.

## Discussion

This is the first study to explore the role of the p16 gene in the development of AA-induced POI by using p16-deficient mice. We found that genetic ablation of the p16 gene had no impact on the ovarian reserve, function or fertility of mice. More importantly, we demonstrated that genetic ablation of the p16 gene did not attenuate ovarian damage or help preserve the fertility of mice challenged by AAs. This study demonstrated for the first time a dispensable role of p16 in POI caused by AAs. Our preliminary findings suggest that targeting p16 alone may not work for preserving the ovarian reserve and fertility of females with POI caused by AAs.

Primordial follicles that initiate folliculogenesis are the foundation of female fertility [24]. A previous study showed that physiologically, p16 was strongly expressed in primordial follicles but weakly expressed in growing follicles, indicating that the downregulation of p16 may initiate the growth of primordial follicles [25]. However, in the present study, we found that p16-deficient mice did not show an apparently reduced number of primordial follicles compared to WT mice. Therefore, p16 may not play a key role in attenuating the growth of primordial follicles under physiological conditions. However, it is possible that p16 may help prevent the overactivation of primordial follicles under stress. It has been reported that AAs induce depletion of primordial follicles by overactivation of primordial germ cells [26]. The upregulation of p16 has been observed in the ovaries of mice with POI induced by AAs [27]. Here, we found that p16 deficiency did not lead to a decreased number of primordial follicles in response to AAs compared to WT mice. Therefore, we concluded that the p16 gene is dispensable for initiating the growth of primordial follicles under either physiological conditions or stress conditions caused by AAs.

Due to the high proliferation activity of granulosa cells, growing follicles are more sensitive to the toxicity of AAs, which can induce cell death by direct intercrossing of DNA double strands. In the present study, a single dose of AAs was given, and the direct killing effect of AAs on growing follicles in WT or p16-deficient mice should be similar at the initial stage. The time point for follicle counts in the present study was 3 months after administering AAs. Undoubtedly, the growing follicles within ovaries treated with AAs at that time point originated from the surviving primordial follicles. The normal function of GCs is crucial for the growth of follicles [28]. Several studies have indicated that the insufficient proliferation of granulosa cells may be associated with the occurrence of POI, indicating that increasing the proliferation of granulosa cells may help to prevent the occurrence of POI [29–32]. It has been observed that p16 is significantly upregulated in granulosa cells in mice with POI [17, 33]. In vitro data indicated that p16 plays a role in decreasing the proliferation of cultured granulosa cells [16, 34]. Therefore, we expected a beneficial role of p16 deficiency in preventing POI by promoting the proliferation of granulosa cells in mice treated with AAs. Unexpectedly, we found that the number of proliferating granulosa cells within growing follicles (normal appearance and nonatretic follicles) was similar between WT and p16-deficient mice treated with or without AAs. In addition, it seems that these growing follicles in WT and KO mice treated with AAs were relatively healthy without obvious apoptosis of granulosa cells. Although “normally developed follicles” existed, the fertility test showed that some WT and KO female mice treated with BUL + CTX were infertile, indicating a poor quality of oocytes. Consistently, p16 deficiency did not increase the number of growing follicles, including antral follicles, or reduce the number of atretic follicles in mice treated with AAs. More importantly, p16 deficiency did not increase the litter size of female mice treated with AAs. These results indicated that p16 is dispensable for the growth of follicles in the ovaries of mice treated with AAs.

As a well-known cell cycle inhibitor and a widely accepted marker for cell senescence, the expression of p16 parallels the progression of ageing and ageing-related disease in multiple organs [35–37]. A large number of studies have demonstrated that targeting p16 could effectively attenuate the progression of ageing and ageing-related disorders [38–40]. However, the expression of p16 is not always detrimental. Issac et al. reported that the expression of p16 was significantly induced in the lung tissue of a mouse model of chronic obstructive pulmonary disease (COPD); however, genetic ablation of p16 could not prevent cellular senescence or alleviate the symptoms of COPD, indicating that p16 alone is dispensable for the development of COPD induced by chronic smoking [41]. The protective role of p16 has also been demonstrated by several studies. Studies conducted by Lv et al. have demonstrated that genetic ablation of p16 promotes liver fibrosis in mice induced by CCL_4_ or a methionine- and choline-deficient diet [42, 43]. Another study showed that deletion of p16 shortened the lifespan and accelerated the disorders of multiple organs of mice bearing homozygous mutations of Pot1b, which plays a critical role in stabilizing the structure of telomeres [44]. A recent study showed that removal of p16^− high^ senescent cells in aged mice is detrimental to the lifespan [45]. Therefore, the detrimental/protective role of p16 may depend on the type of disease and disease context. The present study showed for the first time a dispensable role of p16 in folliculogenesis following treatment with AAs. Other cell cycle inhibitors were also upregulated in the ovaries of mice with POI, such as p21 [46, 47]. Recent studies have highlighted a critical role of p21 in the progression of some diseases. Genetic ablation of p21 has been reported to improve the symptoms of COPD induced by chronic smoking [48]. Similarly, genetic ablation of p21 also prevents liver fibrosis induced by CCL_4_ [49]. In addition, ablation of p21, not p16, could lengthen the lifespan and improve the organ disorder of mice bearing homozygous mutation of Pot1b [44]. Therefore, it is possible that p21 may play a critical role in mediating the occurrence of POI caused by AAs. This will be further investigated in the future by employing p21-deficient mice.

In addition to defective folliculogenesis, ovarian stromal abnormalities, including ovarian stromal fibrosis and damaged vessels, are also common following treatment with chemical drugs [21]. It has been reported that p16 may play a role in promoting CCl4-induced liver fibrosis. Therefore, we speculated that p16 may play a role in regulating fibrosis in the ovary following treatment with AAs. However, in the present study, no significant difference in ovarian fibrosis was observed among groups. Our data indicated that treatment with AAs did not apparently induce the formation of ovarian fibrosis. Patients with cancer showing obvious ovarian fibrosis receive constant treatment with chemical drugs. In contrast, in the present study, the mice received only a single dose of AAs. It is possible that a single dose of AAs may not effectively induce fibrosis. Furthermore, at the age of approximately 18 months (the end of reproductive life), ovarian fibrosis was obvious in naturally aged mice [22]. However, in the present study, the female mice treated with AAs were approximately 6 months old, and most of them were still fertile. The time point set in the present study may not be appropriate for exploring ovarian fibrosis. Consistent with previous studies [23, 50], a reduced ovarian vascularized area of mice treated with AAs was also observed in the present study. However, p16 deficiency had no impact on the ovarian vascularized area caused by AAs. Collectively, we demonstrated that p16 deficiency had no impact on ovarian stromal abnormalities caused by AAs.

## Conclusion

The reproductive span of females is much shorter than their lifespan. The fast ageing of the ovary is the determining factor. The use of chemical drugs further accelerates the ageing of ovaries in females who suffer from cancer. Targeting p16 could attenuate ageing and ageing-related diseases of multiple organs; however, our study demonstrated for the first time that the p16 gene is dispensable for POI induced by AAs. Thus, targeting p16 may not be an effective way to preserve the fertility of females treated with AAs.

## Electronic supplementary material

Below is the link to the electronic supplementary material.

**Supplementary material 1**

## Data Availability

The datasets used and/or analysed during the current study are available from the corresponding author on reasonable request.

## Abbreviations

AAs: Alkylating agents
POI: Premature ovarian insufficiency
BUL: Busulfan
CTX: Cyclophosphamide
PRS: Picrosirius red staining
