# Supplementary material for: Loss of p16 does not protect against premature ovarian insufficiency caused by alkylating agents
Source: BMC Pregnancy Childbirth. 2023 Mar 8;23:151. doi: 10.1186/s12884-023-05476-x (PMC9993597; doi:10.1186/s12884-023-05476-x)
Supplement: Supplementary file 1 — Supplementary material 1 [file 12884_2023_5476_MOESM1_ESM.docx]

**Loss of p16 does not protect against premature ovarian insufficiency caused by alkylating agents**

Fei Liu^1^, Qin Wan^1^, Pengfei Liu^2^, Dengshun Miao^3^, Xiuliang Dai^1^, Li Chen^1^

^1^ The Center for Reproductive Medicine, Changzhou Maternal and Child Health Care Hospital, Changzhou Medical Center, Nanjing Medical University, Changzhou, Jiangsu, China

^2^ Kebiao Medical Testing Center, Jiangsu, China.

^3^ The Research Center for Aging, Affiliated Friendship Plastic Surgery Hospital of Nanjing Medical University, Nanjing Medical University, Nanjing, China.

**Corresponding Authors:** Xiuliang Dai ([daixiuliang@126.com](mailto:daixiuliang@126.com)) and Li Chen ([czrcchenli@126.com](mailto:czrcchenli@126.com)), Center for Reproductive Medicine, Changzhou Maternal and Child Health Care Hospital, Changzhou Medical Center, Nanjing Medical University, Dingxiang Rd, Changzhou 213000, Jiangsu, China


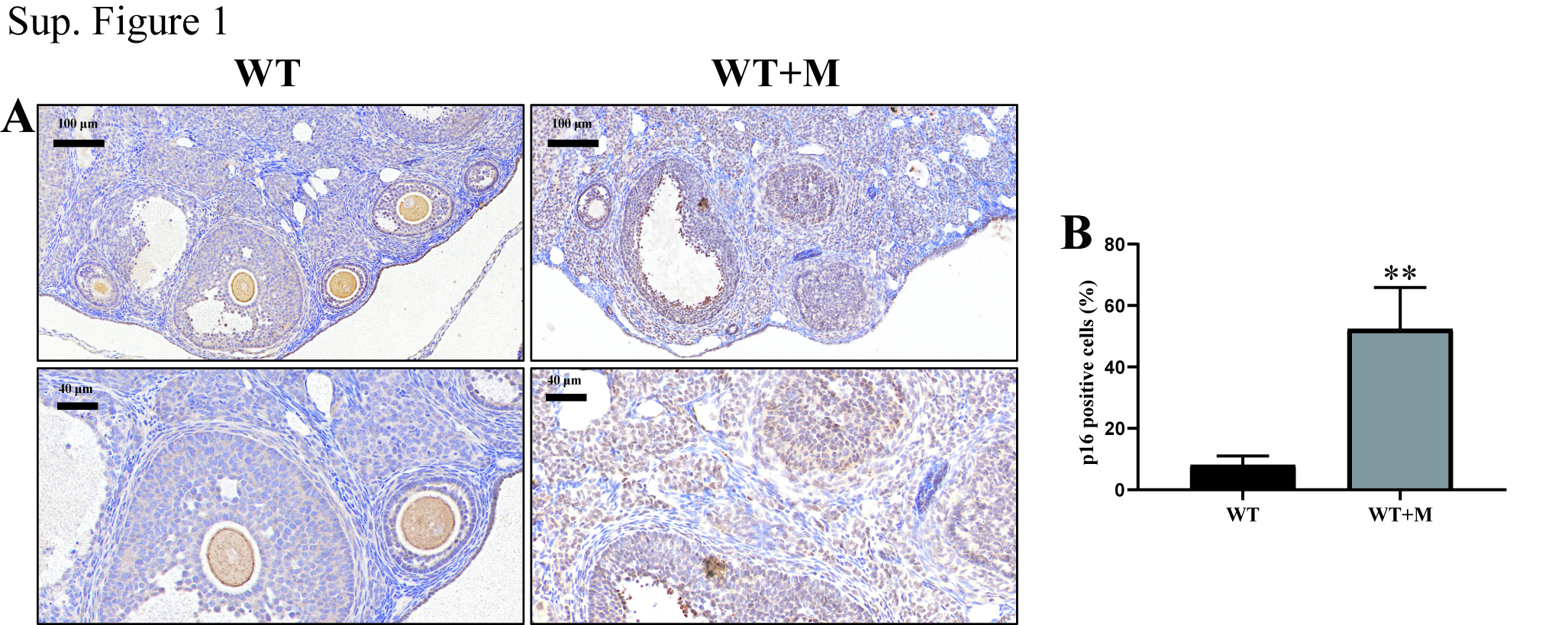


Supplementary figure 1. **p16 was upregulated in the ovaries of mice following treatment with BUL+CTX.** Three months after administering AAs, the ovaries were removed and sectioned. (A) Representative photos of ovarian sections stained with anti-p16 antibody. N= 3 mice in each group. WT: WT mice; WT+M: WT mice treated with BUL+CTX. The upper is 200X; the lower is 400X. (B) The percentage of p16 positive cells. Compared with WT mice: ^**^P < 0.01;


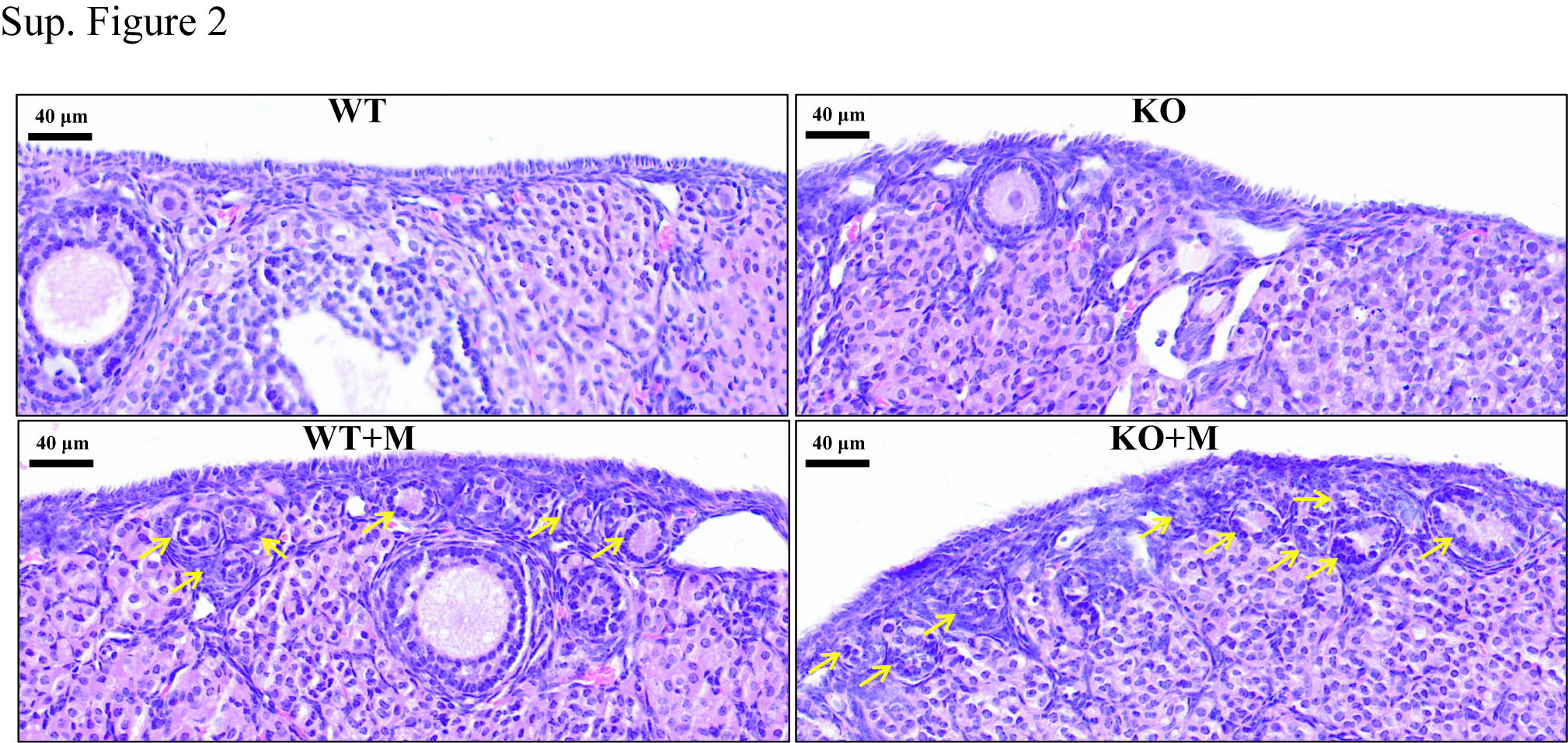


Supplementary figure 2. **Ovaries from mice treated with BUL+CTX showed many follicles without eggs in the ovarian cortex.**  Three months after administering AAs, the ovaries were removed and sectioned. The HE staining of ovarian sections. 400X. WT: WT mice; WT+M: WT mice treated with BUL+CTX; KO mice: untreated p16 KO mice; KO+M: p16 KO mice treated with BUL+CTX. Yellow arrow indicates follicle without egg.
